# A novel approach for the analysis of single-cell RNA sequencing identifies TMEM14B as a novel poor prognostic marker in hepatocellular carcinoma

**DOI:** 10.1038/s41598-023-36650-y

**Published:** 2023-06-28

**Authors:** Ding Ma, Shuwen Liu, Qinyu He, Lingkai Kong, Kua Liu, Lingjun Xiao, Qilei Xin, Yanyu Bi, Junhua Wu, Chunping Jiang

**Affiliations:** 1 State Key Laboratory of Pharmaceutical Biotechnology, National Institute of Healthcare Data Science at Nanjing University, Jiangsu Key Laboratory of Molecular Medicine, Medical School of Nanjing University, Nanjing University, 22 Hankou Road, Nanjing, 210093 Jiangsu China; 2 Jinan Microecological Biomedicine Shandong Laboratory, Shounuo City Light West Block, Qingdao Road 3716#, Huaiyin District, Jinan City, Shandong Province China; 3 Department of Gastroenterology, Third Xiangya Hospital, Central South University, Changsha, Hunan China

**Keywords:** Cancer, Computational biology and bioinformatics

## Abstract

A fundamental goal in cancer-associated genome sequencing is to identify the key genes. Protein–protein interactions (PPIs) play a crucially important role in this goal. Here, human reference interactome (HuRI) map was generated and 64,006 PPIs involving 9094 proteins were identified. Here, we developed a **p**hysical **l**ink **a**nd **c**o-**e**xpression combinatory network construction (PLACE) method for genes of interest, which provides a rapid way to analyze genome sequencing datasets. Next, Kaplan‒Meier survival analysis, CCK8 assays, scratch wound assays and Transwell assays were applied to confirm the results. In this study, we selected single-cell sequencing data from patients with hepatocellular carcinoma (HCC) in GSE149614. The PLACE method constructs a protein connection network for genes of interest, and a large fraction (80%) of the genes (screened by the PLACE method) were associated with survival. Then, PLACE discovered that transmembrane protein 14B (TMEM14B) was the most significant prognostic key gene, and target genes of TMEM14B were predicted. The TMEM14B-target gene regulatory network was constructed by PLACE. We also detected that TMEM14B-knockdown inhibited proliferation and migration. The results demonstrate that we proposed a new effective method for identifying key genes. The PLACE method can be used widely and make outstanding contributions to the tumor research field.

## Introduction

Since the 1970s, increasingly efficient cancer prognosis detection methods and therapeutic approaches have been developed^1–3^, and the list of cancer genes has been growing steadily^4^. There are a large number of differentially expressed genes (DEGs) between cancer tissues and paired adjacent noncancerous tissues, and the key cancer genes often arise from the DEGs, but it is unrealistic to conduct a study on each DEG^5–8^. Fortunately, technological and computational advances in genomics and interactomics have made it possible to screen key genes within human cancer cells^9^.

There are many genome sequencing analysis methods to screen key cancer genes. These methods have the same problem: (1) the accuracy of key gene screening methods needs to be improved^10,11^. (2) Objective regulatory networks for key genes are lacking. There is an urgent need for new key gene screening approaches. The protein–protein interactions (PPIs) are defined as physical links between proteins^12–14^. It is well known that PPIs provide an objective basis, and PPIs could be utilized to screen genes that are consistently associated with survival^15–18^. PPIs have been studied for many years and have been utilized in diverse fields of medicine, such as diagnostics, with a wide range of applications. The revolution brought about by the advent of PPIs has changed the face of human molecular and disease research^19–21^, and it has brought great convenience to human cancer research^22^. PPI networks have vital relationships with gene regulation and function and provide a new way to characterize genes^23^, and many diagnostic markers and therapeutic targets have been identified by PPIs, such as CDK1, SET, and cyclin K^24–26^.

A wide variety of methods have been used to enhance the coverage of PPI identification. Some PPIs are directly obtained by computer simulations, for example, the method of three-dimensional reconstructions of large cellular machinery^27,28^, but there is a deviation between the computer simulation results and real PPIs, resulting in inaccurate PPIs^29^. Some interactions are acknowledged through indirect evidence, such as genetic observations or statistical predictions^30,31^. Genetic observations or statistical predictions provide direction for PPI research, but many genes have the same expression pattern and do not interact with each other^32^, resulting in a waste of research resources.

PPIs are defined based on physical links, and such interactions can only be confirmed if they occur in reality^33^, so comprehensive experimentally validated PPIs may be more trustworthy. Some researchers have experimentally verified the effectiveness of PPIs, but only a small percentage of PPIs have been confirmed by experiments. An incomplete PPI dataset means that the gene interaction network is also incomplete, which leads to significant misinterpretation of gene function. Therefore, we need a comprehensive and accurate PPI dataset. Fortunately, Katja Luck et al. presented a human “all-by-all” reference interactome map (HuRI, the Human Reference Interactome) of human binary protein interactions^34^. Approximately 53,000 PPIs were identified using yeast two-hybrid (Y2H) assays. Other PPIs were reported in the literature by experiments. Finally, the dataset versioned HuRI-union contains 64,006 verified PPIs involving 9094 proteins.

Genome sequencing analysis methods and PPI methods have undeniable deficiencies^35,36^. Genome sequencing analysis methods lack sensitivity and specificity^35^ and cannot be used to build objective regulatory networks^37,38^. PPI methods cannot identify all regulatory relationships between genes^36^. The combination of methods might compensate for the deficiencies. Therefore, in the present study, we combined PPI and genome sequencing analysis to find a better method for screening key genes.

In this study, our aim was to identify key tumor-associated genes that are correlated with the corresponding clinicopathological characteristics and prognosis. We developed a physical link and co-expression combinatory network construction (PLACE) method for the gene of interest, which considered not only the physical links but also the co-expression. The PLACE method allows us to screen the key genes and design a network for the genes of interest. This means that PLACE could be of potential interest to more researchers and will bring more innovative ideas.

## Results

### Hepatocytes and HCC cells were identified based on gene expression patterns and cell markers from tumors

**Figure 1:**
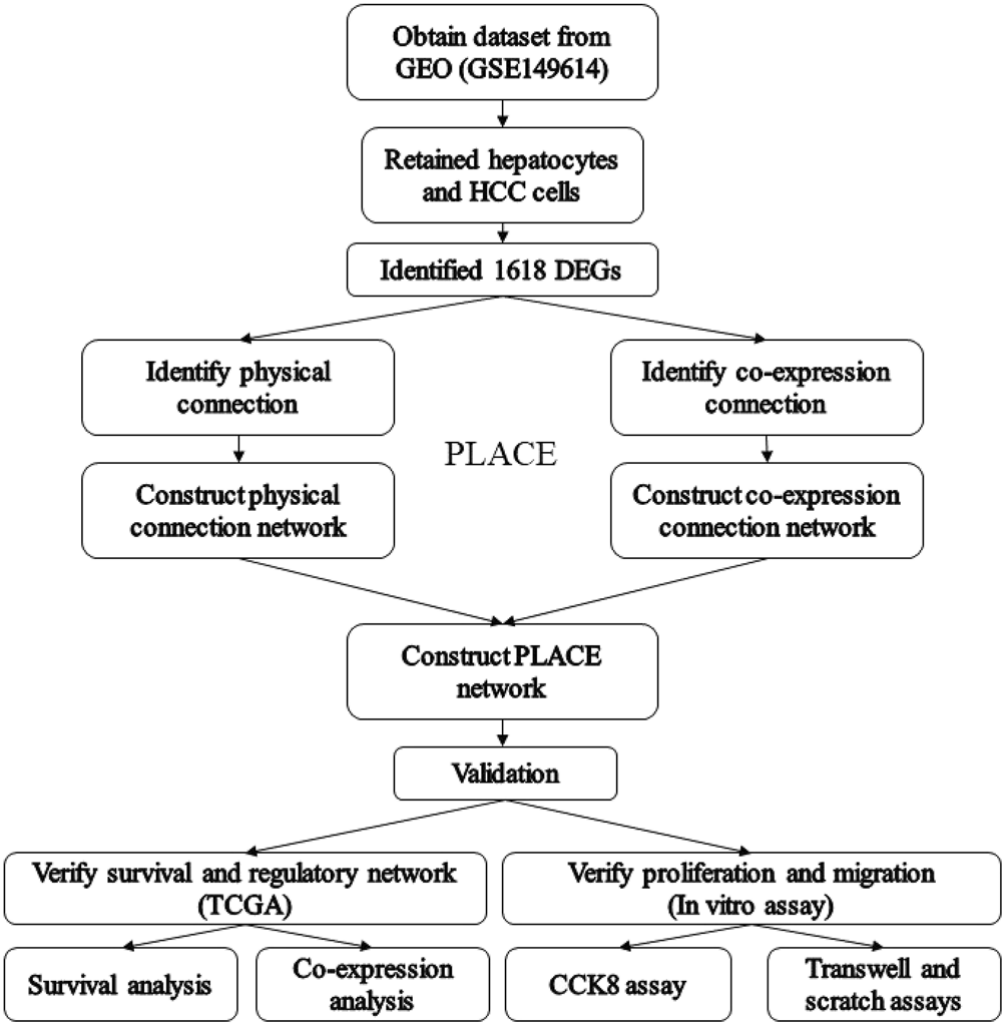
The scheme of PLACE analysis. First, obtain sequencing data from public databases, then select samples of interest from the obtained data, then identified differential genes in obtained data, construct physical linkage networks and co-expression networks by PLCAE, and combine the two networks, Finally, the obtained results are validated by other databases and in vitro assays.

**Figure 2:**
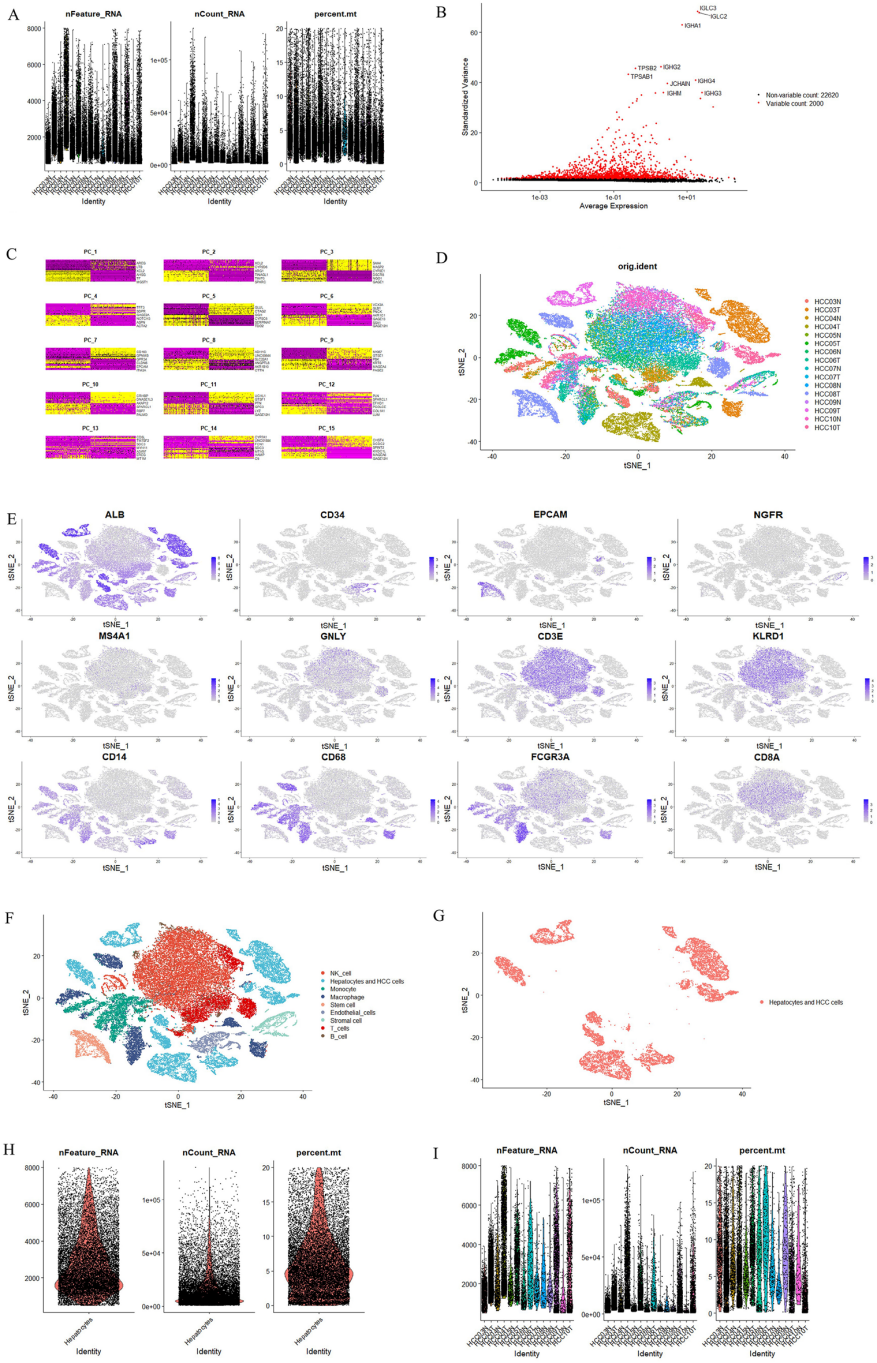
Identification of cell types in HCC samples. (**A**) The violin plots of total gene counts (nCount), number of gene types (nFeature) and the percentage of reads that mitochondrial genes (percent.mt) in each sample of HCC patients from GSE149614. (**B**) A subset of features that shows high variation between cells. Red dot: top 2000 genes that vary significantly in expression between cells, the top 10 highly variable genes were labeled. Black points: Genes with consistent expression level between cells. (**C**) Marker genes extracted from each cell types displayed in 15 Principal Component. (**D**) tSNE plots of 16 liver cancer patients sample between primary tumor (T) and adjacent non-tumor liver (N). Each color marks a cell of one patient to visualize 16 cell classes. (**E**) tSNE that showed the distribution of typical marker (hepatocyte and HCC cell-ALB, endothelial cell-CD34, stem cell-EPCAM, stromal cell-NGFR, B cell-MS4A1, T cell-GNLY, CD3E and CD8A, NK cell-KLRD1, monocyte-CD14 and FCGR3A, macrophage-CD68) representing one cell type in all cells. (**F**) tSNE plots of 9 cell types. (**G**) tSNE plots of hepatocytes and HCC cells. (**H**) Total gene counts (nCount), number of gene types (nFeature) and percentage of mitochondrial genes (percent.mt). Each dot represents a hepatocyte or HCC cell. (**I**) Total gene counts (nCount), number of gene types (nFeature) and percentage of mitochondrial genes (percent.mt). Each dot represents a hepatocyte or HCC cell (grouped by sample).

The study scheme is shown in Fig. 1. HCC cells (T) and hepatocytes (N) were selected from GSE149614 (samples of liver cancer patients) of the GEO database. The data were processed with the Seurat package^39^. We calculated the number of gene types (nFeature^39^) presented in the sample, total gene expression (nCount^39^) and the percentage of reads in the mitochondrial genome (percent.mt^39^), and distinct differences in gene expression levels between T and N were found (Fig. 2A). We next calculated a subset of features that exhibited high cell-to-cell intercellular variation in the dataset (the top 2000 variable genes) (Fig. 2B). Among the 2000 variable genes, we identified 15 principal components, which allowed easy exploration of the primary sources of heterogeneity in a dataset (Fig. 2C). Then, we created an expression matrix of cell-by-gene and conducted dimensionality reduction by T-distributed stochastic neighbor embedding (tSNE) to visualize and explore these datasets (Fig. 2D). We used SingleR to predict and annotate cell type^40^, and then the cell type was confirmed using canonical markers (hepatocyte and HCC cell-ALB, endothelial cell-CD34, stem cell-EPCAM, stromal cell-NGFR, B cell-MS4A1, T cell-GNLY, CD3E and CD8A, NK cell-KLRD1, monocyte-CD14 and FCGR3A, macrophage-CD68) (Table S1) (Fig. 2E). Finally, all cell types were identified and annotated: NK cells, hepatocytes and HCC cells, monocytes, macrophages, stem cells, endothelial cells, stromal cells, T cells, and B cells (Fig. 2F). We retained hepatocytes and HCC cells (Fig. 2G) and calculated nFeature, nCount and percent.mt presented separately in hepatocytes and HCC cells and each sample of hepatocytes and HCC cells for further analysis. (Fig. 2H,I).

### DEGs between HCC cells and hepatocytes

**Figure 3:**
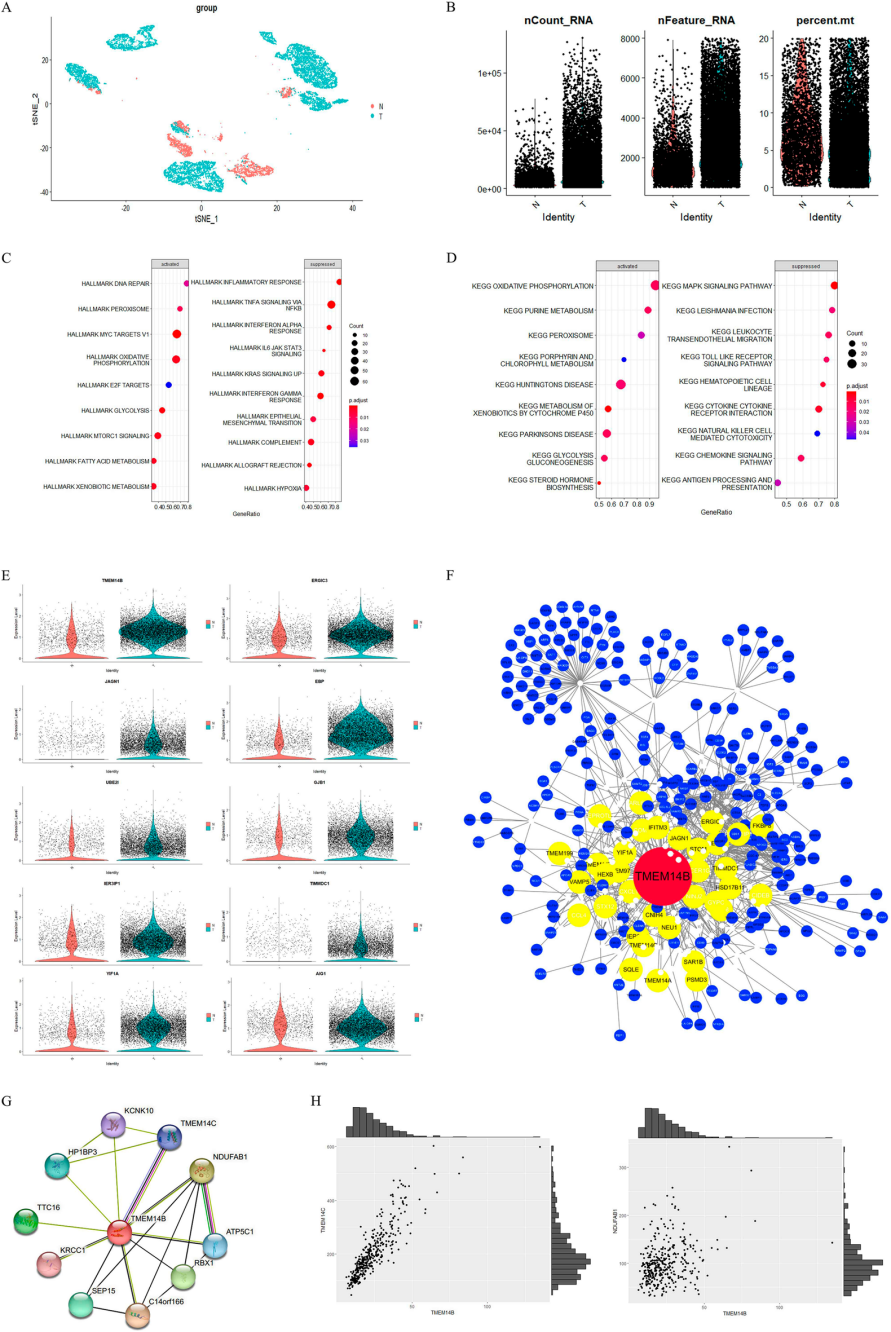
Identification of differential expression genes and construction of protein to protein interaction networks. Hepatocytes and HCC cells of HCC patients from GSE149614 were classified as T (hepatocytes) and N (HCC cells). (**A**) tSNE plots between T and N. (**B**) Total gene counts (nCount), number of gene types (nFeature) and percentage of reads that mitochondrial genes (percent.mt) between T and N. Each dot represents a hepatocyte or HCC cell we selected before. (**C**) Hallmark analysis by clusterProfiler package. Functional enrichment analysis of genome variant genes. Different colors in the p. adjust column on the right indicate different significances, and the sizes of Count indicate the number of genes. (**D**) KEGG pathway analysis by the clusterProfiler package. Functional enrichment analysis of genome variant genes. Different colors in the p. adjust column on the right indicate different significances, and the sizes of Count indicate the number of genes. (**E**) Different expression level of 10 genes screened by PLACE method. Each dot represents a hepatocyte or HCC cell we selected before. (**F**) Protein–protein interaction network of TMEM14B. The yellow circles represent genes which have level1-PPI with TMEM14B. The blue circles represent genes which have level2-PPI with TMEM14B. (**G**) Protein–Protein Interaction Networks of TMEM14B from string. (**H**) The expression between TMEM14B and TMEM14C, NDUFAB1 in TCGA-LIHC.

Hepatocytes and HCC cells were divided into two groups: HCC cells (T) and hepatocytes (N) (Fig. 3A). Besides we calculated nFeature, nCount and percent.mt separately in N and T (Fig. 3B). We then identified 1618 DEGs by comparing cells from HCC with those from hepatocyte (Table S2). We next analyzed DEGs by enrichment in Kyoto Encyclopedia of Genes and Genomes (KEGG) pathways and hallmarks. Hallmark analysis showed that DNA repair, peroxisomes, MYC targets V1, and oxidative phosphorylation were activated (Fig. 3C), and the KEGG results indicated that the DEGs activated in group T were mainly enriched in the oxidative phosphorylation pathway (Fig. 3D). Therefore, follow-up work was performed to help us identify the key genes among the candidate genes.

### Screening candidate genes from DEGs using the PLACE method

The ultimate gene regulatory network requires both physical links and co-expression, as we mentioned earlier. Thus, we analyzed and counted the proportion of DEGs that were significantly correlated (physical links and co-expression) with the target gene in all DEGs. We recalculated the level 1, level 2 and level 3 counts of each DEG of interest using the PLACE method, which has been described in the Methods section. The genes were arranged in descending order by the number of level 1, level 2 and level 3 genes (Table S3). We screened the top10 candidate genes that had the greatest number of PPIs. The expression levels of 10 genes between N and T had significant difference. TMEM14B, ERGIC3, JAGN1, EBP, UBE2I, GJB1, IER3IP1, TIMMDC1, YIF1A and AIG1 were highly expressed in HCC cells (Table 1, Fig. 3E). Finally as an example, PLACE constructed a new network of TMEM14B containing PPIs and co-expression (Fig. 3F), and we verified the relationship of TMEM14B-TMEM14C and TMEM14B-NUFDAB1 by the STRING protein interaction database with the TCGA database (Fig. 3G,H).

**Table 1:** The expression levels of the top 10 candidate genes screened by PLACE.

gene	p_val	avg_log2FC	p_val_adj
TMEM14B	0	0.891007	0
ERGIC3	2.12E−266	0.614997	5.23E−262
JAGN1	1.88E−213	0.354335	4.63E−209
EBP	0	1.286186	0
UBE2I	2.13E−92	0.265321	5.25E−88
GJB1	1.97E−194	0.454651	4.85E−190
IER3IP1	3.55E−174	0.39102	8.73E−170
TIMMDC1	6.80E−183	0.268794	1.67E−178
YIF1A	7.74E−228	0.50631	1.91E−223
AIG1	3.11E−158	0.387093	7.67E−154

### Validation of candidate genes on survival benefit

**Figure 4:**
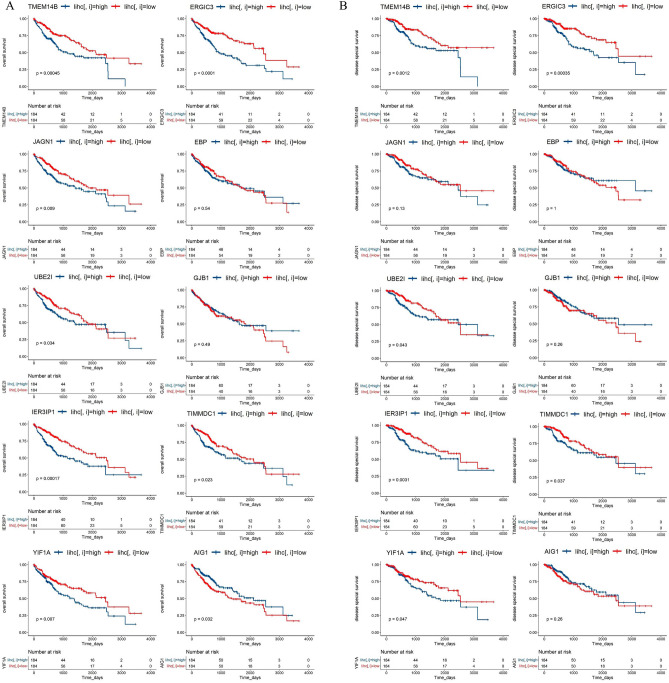
Validation of candidate genes by TCGA-LIHC-TPM data and Survival data. (**A**) Kaplan–Meier curves estimate the overall survival differences between patients with high-expression and low-expression of top10 candidate genes: TMEM14B, ERGIC3, JAGN1, EBP, UBE2I, GJB1, IER3IP1, TIMMDC1, YIF1A and AIG1. (**B**) Kaplan–Meier curves estimate the disease special survival differences between patients with high-expression and low-expression of top10 candidate genes: TMEM14B, ERGIC3, JAGN1, EBP, UBE2I, GJB1, IER3IP1, TIMMDC1, YIF1A and AIG1.

To further verify whether the previously candidate genes can regulate tumor development and thus affect survival, we calculated *p* values for different survival data of each gene by The Cancer Genome Atlas-Liver hepatocellular carcinoma (TCGA-LIHC). Among them, TMEM14B, ERGIC3, JAGN1, BE2I, IER3IP1, TIMMDC1, YIF1A and AIG1 were negatively associated with the overall survival (Fig. 4A), TMEM14B, ERGIC3, UBE2I, IER3IP1 and TIMMDC1 were associated with the disease-specific survival. TMEM14B, ERGIC3, JAGN1 and TIMMDC1 were associated with the disease-free interval. TMEM14B, ERGIC3, UBE2I, IER3IP1 and TIMMDC1 were associated with the progression-free interval (Fig. 4B). A large fraction (80%) of the genes (screened by PLACE method) were associated with survival.

### Construction and validation of the TMEM14B regulatory network

**Figure 5:**
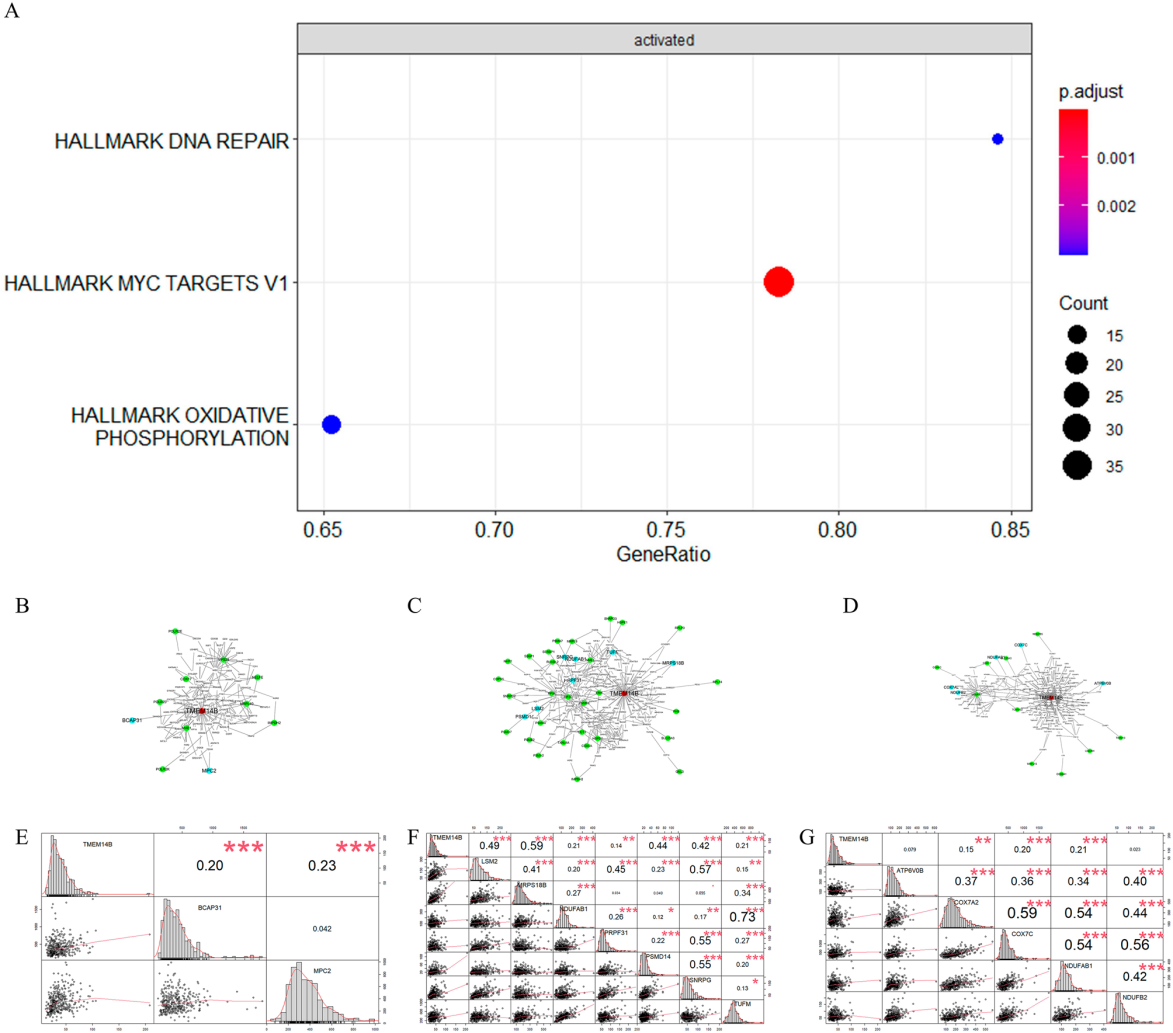
Functional enrichment analysis for the role of TMEM14B. (**A**) Hallmark analysis by clusterProfiler package. Different colors in the p.adjust column on the right indicate different significances, and the sizes of Count indicate the number of genes. (**B**) The regulatory network of TMEM14B in DNA repair. (**C**) The regulatory network of TMEM14B in MYC targets V1. (**D**) The regulatory network of TMEM14B in oxidative phosphorylation. (**E**) The Pearson correlation coefficient between TMEM14B and DNA repair genes. (**F**) The Pearson correlation coefficient between TMEM14B and MYC targets V1 genes. (**G**) The Pearson correlation coefficient between TMEM14B and oxidative phosphorylation genes.

**Figure 6:**
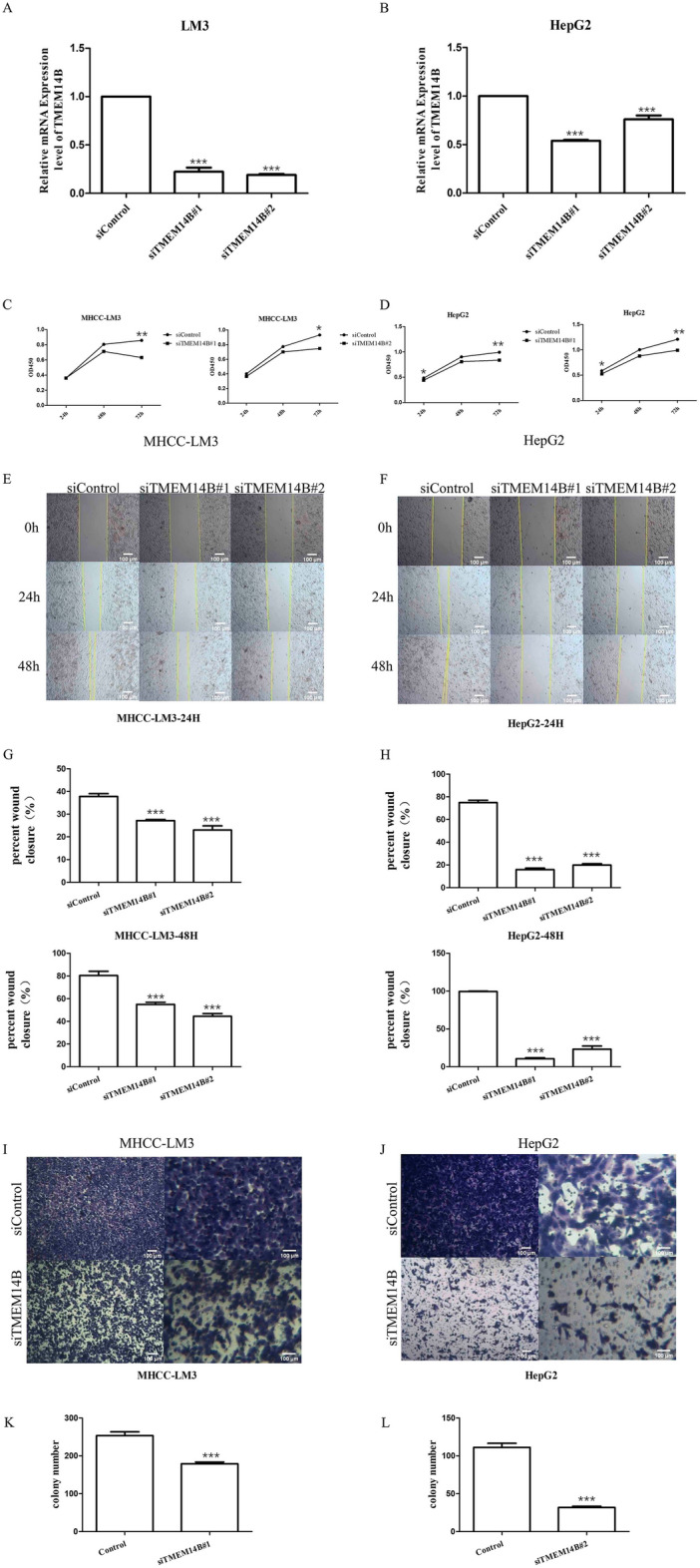
Validation of the role of TMEM14B in vitro. (**A**,**B**) Real-time PCR experiments of LM3 and HepG2 cells transfected with TMEM14B-specific siTMEM14B#1 and siTMEM14B#2 for 24 h. (**C**,**D**) CCK8 assays of LM3 and HepG2 cells transfected with TMEM14B-specific siTMEM14B#1 and siTMEM14B#2 at 24 h, 48 h, and 72 h. (**E**–**H**) Wound healing assays of LM3 and HepG2 cells transfected with TMEM14B-specific siTMEM14B#1 and siTMEM14B#2 at 0 h, 24 h, and 48 h. (**I**–**L**) Transwell experiments of LM3 and HepG2 cells transfected with TMEM14B-specific siRNA for 24 h. *p < 0.01; **p < 0.005; ***p < 0.001.

In the above results, we identified and verified the DEG TMEM14B, which was closely related to the survival of tumor patients. Here, TMEM14B-related genes (Table S4) were screened from the 1618 DEGs by the PLACE method. We next analyzed these TMEM14B-related genes by Hallmark. We then found that DNA repair genes, MYC target V1 genes and oxidative phosphorylation genes were enriched in the TMEM14B-related genes (Fig. 5A). Meanwhile, PLACE was used to construct the TMEM14B regulatory network (Fig. 5B–D). To further verify whether TMEM14B could regulate DNA repair genes, MYC targets V1 genes and oxidative phosphorylation genes, the interrelationships among the genes (TMEM14B-DNA repair genes, TMEM14B-MYC targets V1 and TMEM14B-oxidative phosphorylation) were validated by TCGA-LIHC. We discovered that 8 of the 11 TMEM14B-DNA repair gene interactions were detected by our method and confirmed by the Pearson test in the TCGA-LIHC cohort, 34 of the 46 TMEM14B-MYC target V1 gene interactions were detected by our method and confirmed by the Pearson test in the TCGA-LIHC cohort, and 11 of the 15 TMEM14B-oxidative phosphorylation gene interactions were detected by our method and confirmed by the Pearson test in the TCGA-LIHC cohort (Fig. 5E–G, Tables S5–S7). To confirm the carcinogenic role of TMEM14B, we knocked down TMEM14B in HepG2 and MHCC-LM3 cells using siRNA. Cell proliferation was evaluated using a CCK-8 assay at 24 h, 48 h and 72 h. The results showed that TMEM14B knockdown inhibited the proliferation of HepG2 and LM3 cells (Fig. 6A–D). Cell migration was evaluated using Transwell and scratch assays. TMEM14B knockdown inhibited the migration of HepG2 and LM3 cells (Fig. 6E–L). This result emphasized another advantage of the PLACE method: we can construct a PPI and co-expression network for each protein that is useful for studying genes of interest.

## Discussion

In the present study, we proposed a new method, PLACE. In the PLACE method, the input of PPI interactions, expression matrix and potential gene list were needed, and then the co-expression and physical link (level 1, level 2, level 3) network of each potential gene was accordingly constructed. After sorting by PLACE, potential genes that ranked in the top 5, 10, 20, 30, 40 or 50 of the list were selected as key genes.

PPI interactions stem from computational prediction, from knowledge about intricate connections and information transfer between molecules within organisms, and from interactions aggregated from other primary databases^41,42^. There are several published databases of PPIs, such as The Search Tool for the Retrieval of Interacting Genes/Proteins (STRING) database and BioGRID database^16,43^. For these databases, while comprehensive, no uniform standard definitions were used for the PPIs. Therefore, these databases were not used in our study, but the Human Reference Interactome database (HuRI) was used. Benefiting from the Center for Cancer Systems Biology at Dana-Farber Cancer Institute, a human “all-by-all” reference interactome map of human binary protein interactions was successfully constructed. Currently, 64,006 PPIs involving 9094 proteins have been identified using the Y2H assay^22^. The Y2H assay is the least laborious, low-cost, high-precision direct PPI screening method available to date^44^. PLACE can further dissect key genes based on HuRI PPI interaction data.

The expression matrix and potential gene list from a total of 13,736 cells (10,672 cancer tissue-derived cells and 3064 paired adjacent noncancerous tissue-derived cells) were picked from the scRNA-seq, and we annotated hepatocytes and HCC cells using canonical markers, such as ALB^45^. The exclusion of other cell types by design implied that our results have no bearing for immune cells, stromal cells, etc., so we only focused on the hepatocytes and HCC cells themselves. Then, we identified a number of genes that were differentially expressed between cancer tissues and paired adjacent noncancerous tissues.

In this article, the PPI interactions, expression matrix and potential gene list were processed using PLACE. Next, TMEM14B was identified as the most significant prognostic key gene. Survival is the key to prognosis for tumors; thus, we thought that differentially expressed key genes strongly correlated with survival determine the different prognoses of cancer patients^46,47^, so we analyzed the correlation between gene expression level and survival to evaluate the importance of a gene.

In the present study, TMEM14B regulatory hallmarks, such as DNA repair, MYC targets V1 and oxidative phosphorylation, were found by analyzing the GSE149614 dataset in PLACE, and the results were proven by TCGA. For TMEM14B, biological experiments were conducted, indicating its critical role in the pathogenesis of multiple carcinomas. In conclusion, we have found a new method for discovering critical genes. The role of TMEM14B in tumors is not clear, and this study revealed its prognostic role and regulatory network in HCC for the first time. The results proved that PLACE makes it possible to accurately connect key genes to the regulatory pathway.

## Materials and methods

### PLACE method

The PPI network was constructed using the HuRI-Union dataset. The PPIs in HuRI were identified by yeast two-hybrid (Y2H) assay or curated literature. For ease of use, we redefined 3 relationships (between any two proteins A and B). Level 1: Proteins A and B in direct contact and interaction—protein A-protein B; level 2: Proteins A and B in indirect contact with an interval of protein X—protein A-protein X-protein B; level 3: Proteins A and B in indirect contact with an interval of two proteins X1 and X2—protein A-protein X1-protein X2-protein B. We calculated the level 1 counts, level 2 counts and level 3 counts for each DEG. Apart from this, we then examined the relationship between each DEG, and Pearson’s coefficient was calculated for all genes. We retained the level 1 counts, level 2 counts and level 3 counts based on correlation values r > 0.5 and *p* < 0.05, and the network was visualized with Cytoscape software. The genes were arranged in descending order by the number of level 1, level 2 and level 3 genes.

### Data processing

We downloaded GSE149614 scRNA-seq submitted by Yiming Lu et al. from the Gene Expression Omnibus database^48^. A total of 13,736 cells (10,672 cancer tissue-derived cells and 3064 paired adjacent noncancerous tissue-derived cells) were selected from the scRNA-seq.

We downloaded TCGA-LIHC-FPKM data from The Cancer Genome Atlas Program. We subsequently converted FPKM values to TPM (transcripts per million) using TPM = [FPKM/FPKMsum] * 10^6^. We also downloaded survival data from The Cancer Genome Atlas Program (https://xena.ucsc.edu/public/).

We downloaded the HuRI-union dataset submitted by Luck et al. (64,006 PPIs involving 9094 proteins were identified)^34^.

### Single-cell RNA sequencing data analysis: dimensionality reduction and clustering

After preliminary screening of 13,736 cells (10,672 cancer tissue-derived cells and 3064 paired adjacent noncancerous tissue-derived cells), the cutoff criteria iare that the percentage of mitochondria is less than 20%, and the expression matrix of cells was processed using R software (Seurat package). Following data normalization (NormalizeData Function) and scaling (ScaleData Function), principal component analysis (PCA) was conducted using genes with highly variable expression. Seurat graph-based clustering was then applied to visualize the identified clusters in tSNE plots (RunTSNE Function).

### Single-cell RNA sequencing data analysis: cell type annotation

The cell types were annotated according to a sample reference dataset (HumanPrimaryCellAtlasData) with known labels given via the SingleR package, which assigns these labels to cells from GSE149614 based on the similarity of their expression profiles and confirmed according to the list of marker genes (Table S1). We visualized the marker genes in clustering plots by the FeaturePlot function.

### Single-cell RNA sequencing data analysis: biomarker genes that showed differential expression between cancer cell-derived hepatocytes and HCC cells

Hepatocytes and HCC cells were selected from the pool of single cells (subset Function). We performed differential gene expression analyses on cancer tissue-derived cells and paired adjacent noncancerous tissue-derived cells. Differentially expressed genes (DEGs) were then identified by differential gene expression analysis. The Wilcoxon test (adjusted *P* value < 0.05) and a log_e_ (FC) greater than 0.25 were used to test for significance^39^.

### Gene enrichment analysis

With the help of the clusterProfiler package and GSEA dataset, hallmark enrichment and KEGG pathway enrichment were performed using the hallmark gene set (http://www.gsea-msigdb.org/gsea/msigdb/index.jsp) and KEGG database (https://www.genome.jp/kegg/).

### Validation using TCGA RNA-seq data

To determine the value of the prognostic gene signature in prognosis at the RNA level, TCGA-LIHC TPM data and survival data were used for validation. Survival was analyzed using Kaplan–Meier survival analysis. Overall survival (OS) and disease-specific survival (DSS) of HCC patients with the gene of interest were assessed and compared between the long-survival and short-survival groups.

### Validation in human HCC cell lines

To determine the functions of TMEM14B, we knocked down TMEM14B expression using siRNA in human HCC cell lines (LM3 and HepG2).

- siTMEM14B#1: sense5′-3′ GUGCUUACCAGCUGUAUCATT,
- siTMEM14B#2 sense5′-3 GCCUGUAGGUUUAAUUGCATT.

### Cell counting kit 8 (CCK8) assay

For the Cell Counting Kit-8 (CCK-8) assay, LM3 and HepG2 cells in DMEM containing 10% FBS were seeded into 96-well plates at a concentration of 1 × 10^4^ cells per well and incubated for 24 h, 48 h and 72 h. CCK-8 solution (10 μl/well) was added to the 96-well plates and incubated for 1 h to detect the viability of LM3 and HepG2 cells. The light absorbance values at 450 nm were measured in a microplate reader (Bio-Rad, Hercules, CA, United States), and cell viability was determined.

### Wound-healing assay

A culture insert (Ibidi, Munich, Germany) was used to generate a wound of 500 μm. The insert was placed on 24-well plates; then, 3 × 10^5^ cells were seeded in each culture insert and incubated for 24 h. After removing the culture insert, the cells were allowed to grow in medium without FBS for 24 h. The original area and migration area were measured using ImageJ software, and the wound closure rates are shown according to the ratio of the migration area to the original area. Each treatment was performed in triplicate wells, and three independent experiments were repeated.

### Transwell assay

Transwell migration assays were performed using a 6.5-mm transwell insert with an 8.0-μm pore polycarbonate membrane (Merck Millipore, Burlington, MA, United States). A total of 300 μl of cell suspension containing 3 × 10^5^ cells without FBS was added to the upper chamber, and 800 μl of medium containing 10% FBS was added to the lower chamber. After incubation for 24 h, cells in the lower chamber were fixed with 4% paraformaldehyde for 15 min and stained with crystal violet for 15 min. Images of each chamber were captured randomly for cell counting. Three independent experiments were repeated.

## Supplementary Information

Supplementary Legends.

Supplementary Table S1.

Supplementary Table S2.

Supplementary Table S3.

Supplementary Table S4.

Supplementary Table S5.

Supplementary Table S6.

Supplementary Table S7.

## Data Availability

The authors confirm that the data supporting the findings of this study are available within Gene Expression Omnibus database (https://www.ncbi.nlm.nih.gov/geo/query/acc.cgi?acc=GSE149614) and The Cancer Genome Atlas Program (https://xena.ucsc.edu/public/).
