# Supplementary material for: A novel approach for the analysis of single-cell RNA sequencing identifies TMEM14B as a novel poor prognostic marker in hepatocellular carcinoma
Source: Sci Rep. 2023 Jun 28;13:10508. doi: 10.1038/s41598-023-36650-y (PMC10307879; doi:10.1038/s41598-023-36650-y)
Supplement: Supplementary file 1 — Supplementary Legends. [file 41598_2023_36650_MOESM1_ESM.docx]

Table S1 Canonical markers of different cell types

Table S2 1618 DEGs between primary tumor tissue and adjacent non-tumor tissue

Table S3 The DEGs were arranged by the PLACE method

Table S4 TMEM14B-related DEGs

Table S5 Correlation between TMEM14B and DNA repair gene was statistically evaluated using Pearson correlation coefficient.

Table S6 Correlation between TMEM14B and MYC target V1 gene was statistically evaluated using Pearson correlation coefficient.

Table S7 Correlation between TMEM14B and oxidative phosphorylation gene was statistically evaluated using Pearson correlation coefficient.
