# Supplementary material for: A novel approach for the analysis of single-cell RNA sequencing identifies TMEM14B as a novel poor prognostic marker in hepatocellular carcinoma
Source: Sci Rep. 2023 Jun 28;13:10508. doi: 10.1038/s41598-023-36650-y (PMC10307879; doi:10.1038/s41598-023-36650-y)
Supplement: Supplementary file 6 — Supplementary Table S5. [file 41598_2023_36650_MOESM6_ESM.docx]

| Gene name | pvalue | rvalue |
| --- | --- | --- |
| TMEM14B | 0 | 1 |
| BCAP31 | 0.000101 | 0.201064 |
| MPC2 | 1.02E-05 | 0.227512 |
| COX17 | 0.000129 | 0.198002 |
| IMPDH2 | 4.50E-05 | 0.210722 |
| MRPL40 | 2.67E-06 | 0.241598 |
| NELFE | 3.29E-37 | 0.598372 |
| NME1 | 5.04E-08 | 0.279004 |
| POLR2E | 0.494167 | 0.035702 |
| POLR2J | 0.477229 | 0.037114 |
| POLR2K | 4.09E-06 | 0.237192 |
| VPS28 | 0.739736 | 0.017351 |

Table S5 Correlation between TMEM14B and DNA repair gene was statistically evaluated using Pearson correlation coefficient.
