# Supplementary material for: A novel approach for the analysis of single-cell RNA sequencing identifies TMEM14B as a novel poor prognostic marker in hepatocellular carcinoma
Source: Sci Rep. 2023 Jun 28;13:10508. doi: 10.1038/s41598-023-36650-y (PMC10307879; doi:10.1038/s41598-023-36650-y)
Supplement: Supplementary file 7 — Supplementary Table S6. [file 41598_2023_36650_MOESM7_ESM.docx]

| Gene name | pvalue | rvalue |
| --- | --- | --- |
| TMEM14B | 0 | 1 |
| LSM2 | 5.22E-24 | 0.493132 |
| MRPS18B | 6.76E-36 | 0.589439 |
| NDUFAB1 | 3.52E-05 | 0.2136 |
| PRPF31 | 0.007708 | 0.138515 |
| PSMD14 | 4.27E-19 | 0.442184 |
| SNRPG | 3.68E-17 | 0.419499 |
| TUFM | 3.14E-05 | 0.214902 |
| CCT3 | 5.03E-14 | 0.378643 |
| COPS5 | 2.54E-07 | 0.26446 |
| COX5A | 0.022727 | 0.118571 |
| EIF3D | 1.37E-13 | 0.372491 |
| ERH | 7.58E-08 | 0.275412 |
| GNL3 | 5.72E-10 | 0.315449 |
| HSPD1 | 2.30E-15 | 0.396808 |
| HSPE1 | 4.84E-10 | 0.316717 |
| IMPDH2 | 4.50E-05 | 0.210722 |
| MRPL9 | 2.85E-15 | 0.395574 |
| NHP2 | 1.15E-13 | 0.373611 |
| NME1 | 5.04E-08 | 0.279004 |
| PHB | 2.49E-26 | 0.514536 |
| PPIA | 1.45E-10 | 0.325699 |
| PSMA2 | 0.381739 | 0.045666 |
| PSMA4 | 4.78E-09 | 0.298802 |
| PSMA7 | 5.03E-07 | 0.25804 |
| PSMB2 | 1.03E-07 | 0.2727 |
| PSMB3 | 0.000163 | 0.195072 |
| PSMD7 | 2.57E-07 | 0.26434 |
| RPL14 | 0.008767 | 0.136269 |
| RPLP0 | 0.006818 | 0.140625 |
| RUVBL2 | 6.38E-06 | 0.232513 |
| SERBP1 | 7.37E-12 | 0.34667 |
| SLC25A3 | 0.000607 | 0.17765 |
| SNRPD2 | 0.136666 | 0.077625 |
| SNRPD3 | 6.62E-12 | 0.347392 |
| SSBP1 | 1.74E-06 | 0.245916 |
| TXNL4A | 1.37E-06 | 0.248308 |

Table S6 Correlation between TMEM14B and MYC target V1 gene was statistically evaluated using Pearson correlation coefficient.
