# Supplementary material for: A novel approach for the analysis of single-cell RNA sequencing identifies TMEM14B as a novel poor prognostic marker in hepatocellular carcinoma
Source: Sci Rep. 2023 Jun 28;13:10508. doi: 10.1038/s41598-023-36650-y (PMC10307879; doi:10.1038/s41598-023-36650-y)
Supplement: Supplementary file 8 — Supplementary Table S7. [file 41598_2023_36650_MOESM8_ESM.docx]

| Gene name | pvalue | rvalue |
| --- | --- | --- |
| TMEM14B | 0 | 1 |
| ATP6V0B | 0.130411 | 0.078882 |
| COX7A2 | 0.00438 | 0.148018 |
| COX7C | 0.000134 | 0.197527 |
| NDUFAB1 | 3.52E-05 | 0.2136 |
| NDUFB2 | 0.653536 | 0.023443 |
| COX17 | 0.000129 | 0.198002 |
| COX6A1 | 0.067082 | 0.095431 |
| COX6C | 0.626429 | -0.02542 |
| MRPL15 | 0.000208 | 0.191924 |
| NDUFB5 | 2.80E-14 | 0.382187 |
| NDUFB7 | 0.002814 | 0.155099 |
| SLC25A3 | 0.000607 | 0.17765 |
| TIMM13 | 0.005516 | 0.144207 |
| TOMM22 | 6.13E-09 | 0.296789 |
| UQCRH | 2.73E-07 | 0.263775 |

Table S7 Correlation between TMEM14B and oxidative phosphorylation gene was statistically evaluated using Pearson correlation coefficient.
